# NRDR inhibits estradiol synthesis and is associated with changes in reproductive traits in pigs

**DOI:** 10.1002/mrd.23080

**Published:** 2018-11-23

**Authors:** Ying Liu, Yalan Yang, Wentong Li, Yanmin Zhang, Yanzhao Yang, Hua Li, Zhaoyu Geng, Hong Ao, Rong Zhou, Kui Li

**Affiliations:** ^1^ The State Key Laboratory for Animal Nutrition, Institute of Animal Science, Chinese Academy of Agricultural Sciences Beijing China; ^2^ College of Life Science and Engineering, Foshan University, Foshan Guangdong China; ^3^ College of Animal Science and Technology, Anhui Agricultural University Hefei Anhui China

**Keywords:** cumulus cells, NADPH‐dependent retinol dehydrogenase/reductase ( *NRDR*), pig, reproduction traits, steroid hormone biosynthesis

## Abstract

Cumulus cells secreting steroid hormones have important functions in oocyte development. Several members of the short‐chain dehydrogenase/reductase (SDR) family are critical to the biosynthesis of steroid hormones. NADPH‐dependent retinol dehydrogenase/reductase ( *NRDR*), a member of the SDR superfamily, is overexpressed in pig breeds that also show high levels of androstenone. However, the potential functions and regulatory mechanisms of *NRDR* in pig ovaries have not been reported to date. The present study demonstrated that *NRDR* is highly expressed in pig ovaries and is specifically located in cumulus granulosa cells. Functional studies showed that *NRDR* inhibition increased estradiol synthesis. Both pregnant mare serum gonadotropin and human chorionic gonadotropin downregulated the expression of *NRDR* in pig cumulus granulosa cells. When the relationship between reproductive traits and single‐nucleotide polymorphisms (SNPs) of the *NRDR* gene was examined, we found that two SNPs affected reproductive traits. SNP rs701332503 was significantly associated with a decrease in the total number of piglets born during multiparity, and rs326982309 was significantly associated with an increase in the average birth weight during primiparity. Thus, *NRDR* has an important role in steroid hormone biosynthesis in cumulus granulosa cells, and *NRDR* SNPs are associated with changes in porcine reproduction traits.

## 1 INTRODUCTION

In mammals, oocyte development is carefully regulated by the surrounding somatic cells (Hoffmann & Maser, 2007; Park et al., 2004; Robinson et al., 2012). Two populations of granulosa cells are important in this regulation as follows: cumulus cells that associate with oocytes and mural cells that line the outer limits of the follicle. Previous reports have shown that cumulus cells secrete steroid hormones in humans (Chian, Ao, Clarke, Tulandi, & Tan, 1999; Teves et al., 2006), mice (Vanderhyden & Tonary, 1995), and pigs (Yamashita, Shimada, Okazaki, Maeda, & Terada, 2003). Progesterone (P4) is secreted by the cumulus‐oocyte complex (COC) during in vitro maturation, and the level of progesterone secretion increases when the COC is stimulated with luteinizing hormone (LH) and follicle‐stimulating hormone or forskolin in pigs (Coskun, Uzumcu, Lin, Friedman, & Alak, 1995; Racowsky, 1985; Shimada & Terada, 2002; Xia et al., 1994), mice (Vanderhyden & Tonary, 1995), and cattle (Armstrong, Xia, de Gannes, Tekpetey, & Khamsi, 1996). Although there are a number of publications focused on the regulation of progesterone secretion in oocytes, the regulation of estradiol (E2) synthesis and secretion is poorly understood.

The NADPH‐dependent retinol dehydrogenase/reductase (*NRDR*) gene, also known as dehydrogenase/reductase short‐chain alcohol dehydrogenase/reductase family member 4 (*DHRS4*), is located on the 14q11.2 chromosome (GenBank accession number AB045131) in the human genome, and it was initially puriﬁed from rabbit liver (Huang & Ichikawa, 1997). This gene is a member of the short‐chain dehydrogenase/reductase (SDR) superfamily, and it shares the basic structural and functional features of the SDR superfamily (Du et al., 2004; Usami et al., 2003). The SDR superfamily is one of the largest and oldest protein families, and it is involved in the metabolism of a large variety of compounds, including steroid hormones, prostaglandins, retinoids, lipids, and xenobiotics (Bray, Marsden, & Oppermann, 2009; Persson et al., 2009).

*NRDR* is widely distributed in human organs and tissues. Because of the high activity of retinal reductase at physiological pH compared to other enzymes in the SDR superfamily (G. Lee & Bendayan, 2004), *NRDR* plays an important role in maintaining the homeostasis of retinal and retinoic acid. The *NRDR* gene cluster is located in an area of segmental duplications, and the expression of this cluster is downregulated in some common human cancers (Lu et al., 2007). Human *DHRS4* may have a novel role in human endocrinology. Expression of *DHRS4* is induced via peroxisome proliferator‐activated receptor (PPARα) activation. PPARα regulates various genes that control fatty acid catabolism, gluconeogenesis, ketone body synthesis, hormone synthesis, and cholesterol metabolism (Michalik et al., 2006). In other species, including pig, mouse, and dog, NRDR protein is also efficient at reducing retinal metabolism (Endo et al., 2007; Lei, Chen, Zhang, & Napoli, 2003; Usami et al., 2003). The human ortholog of *NRDR*, initially termed peroxisomal 2,4‐dienoyl CoA reductase‐related protein (Fransen, Van Veldhoven, & Subramani, 1999), is significantly active in reducing various aromatic ketones and alpha‐dicarbonyl compounds, including 3‐ketosteroids and cytotoxic 9,10‐phenanthrenequinone (Endo et al., 2009; Kisiela, El‐Hawari, Martin, & Maser, 2011; Matsunaga et al., 2008). *NRDR* is an important enzyme involved in the reductive metabolism of carbonyl groups, and it contributes to essential physiological roles in the metabolism of steroid hormones and retinoids in different species (M. S. Song, Chen, Zhang, & Napoli, 2003). *NRDR* is highly expressed in the pig testis, and it has been implicated in the production of steroid hormones (Moe et al., 2007; Rolland et al., 2009). Thus far, no studies have been reported on the expression and function of *NRDR* in the ovary. Our previous transcriptome sequencing revealed that *NRDR* expression varies in different stages of pregnancy in porcine ovaries (unpublished).

The primary aim of the present study was to determine *NRDR* expression and related functions in pig ovary. The results first demonstrated that *NRDR* is highly expressed in pig ovary and that it downregulates estradiol synthesis in pig cumulus granulosa cells. A relationship between reproductive traits and polymorphisms of the *NRDR* gene (*DHRS4*) was also demonstrated. One SNP was significantly associated with the total number of piglets born during multiparity, and a second SNP was significantly associated with changes in the average birth weight (ABW) during primiparity. The results describing the *NRDR* and E2 signaling pathway in cumulus cells increases the understanding of ovary physiology and reveals potential targets for pharmacological interventions. The results pertaining to SNPs may also be useful for marker‐assisted selection and genomic selection strategies for genetic improvement programs in pigs.

## 2 MATERIALS AND METHODS

### 2.1 Animals

All procedures performed on animals followed the guidelines of the China Council on Animal Care and were approved by the Chinese Association for Laboratory Animal Sciences. Heart, liver, spleen, lung, kidney, ovary, uterus, and oviduct tissues were collected from adult Yorkshire and Meishan pigs on postnatal days 180 and 300 for spatial expression analysis. Three replicates of each tissue were collected from both Yorkshire and Meishan pigs at each stage. Samples were harvested immediately after slaughter, frozen in liquid nitrogen, and stored at −80°C. For the investigation of allele frequency, ear tissue (*n* = 234) was collected from a crossbred population derived from Yorkshire and Chinese Yimeng black pigs raised by the Lansi Breeding Corporation (Rizhao, Shandong, China). The total number born (TNB), total litter weight (TLW), and ABW of animals were recorded during consecutive years from 2005 to 2010. The average parity of pigs used in association analysis was five. The records of TNB, TLW, and ABW were available for each individual and used for association analysis with *DHRS4* SNPs.

### 2.2 Cell culture

The ovaries used for in vitro experiments were obtained from Yorkshire pigs from a local slaughterhouse and transported to the laboratory within 2 hr of harvest. The ovaries were maintained at 37°C in a sterile physiological saline solution (0.9% NaCl, 100 IU/mL penicillin, and 100 IU/mL streptomycin). For cell culture, the follicular ﬂuid was aspirated from 3 to 5 ml follicles using a 20 ml syringe and centrifuged at 500 g for 5 min to collect the COC. The COCs surrounded by a compact cumulus mass with evenly granulated cytoplasms were picked out using a mouth pipette under a microscope. Serum‐free TCM‐199 (Gibco, Burlington, ON, Canada) buffered with 10 mM HEPES and 26 mM bicarbonate was used for washing COCs. COCs were pipetted into 1.5 ml tubes, and 150 IU hyaluronidase was added and incubated for 3 min at 37°C. Oocytes were then picked out using a mouth pipette, and cumulus cells were collected by centrifugation at 1,000 g for 3 min at room temperature. Cumulus cells were then collected from the bottom of the centrifuge tube. The cumulus cells were washed three times with serum‐free DMEM/F12 culture medium (Invitrogen, Carlsbad, CA). Cells were dispersed by pipetting up and down several times. Cell viability was determined using Trypan blue dye (Sigma‐Aldrich, MO). Cells were seeded at a concentration of 1 × 10^5^ cells per well in 12‐well plates containing 1 mL of DMEM/F12 supplemented with 10% fetal bovine serum (Invitrogen, CA). Cells were cultured in a highly humidified atmosphere of 95% air and 5% CO_2_ at 37°C. Porcine cumulus cells were cultured in DMEM/F12 medium with 10% fetal calf serum for 72 hr before further treatment, and the culture medium was refreshed every 24 hr.

### 2.3 Cells transient transfection and treatment

An *NRDR* siRNA kit was purchased from RiboBio (Guangzhou, China), which contained three siRNAs for *NRDR* and an NC‐siRNA. The transient transfections were performed as previously described (Liu et al., 2015; Wu et al., 2010). Cultured cells were treated with 10 IU/ml hCG or PMSG for 0 (CON), 3, 6, 12, and 24 hr.

### 2.4 Analysis of cell cycle by flow cytometry

Forty‐eight hours after transfection, cells were fixed in 70% (v/v) ethanol overnight at −20°C. Following incubation in 50 μg/ml propidium iodide (Sigma‐Aldrich, MO) containing 100 μg/ml RNase A (Qiagen, Beijing, China) and 0.2% (v/v) TritonX‐100 (Sigma‐Aldrich, MO) for 30 min at 4°C, 20,000 cells of each sample were analyzed in a FACSCalibur flow cytometer (BD Biosciences, CA) using ModFit software. Statistical analysis of the cell ratio for each cell cycle was performed.

### 2.5 Cell viability assay and statistical analysis

Twenty‐four hours after transfection, cells were cultured in 96‐well plates (1 × 10^4^ cells/well). Desired drugs and compounds were added to the wells separately immediately after cells were seeded and cultured with the cells for 24 hr. Cell viability was measured by fluorescence chemistry using a CCK8 kit (Lianke Bio, Beijing, China) with a spectrophotometer (Multiskan MK3; Thermo Fisher Scientific, Rockford, IL) at an optical density (OD) of 450 nm.

Each experimental condition was repeated three times with up to five multiple wells each time. Data are presented as the mean ± standard error, and statistical significance was calculated by Student’s *t* test in Excel. *P* < 0.05 was considered significantly different.

### 2.6 Isolation of RNA and real‐time quantitative PCR (RT‐qPCR)

Tissue samples (50–100 mg), which were stored at −80°C, were homogenized in 1 ml of TRIZOL (Invitrogen, CA). Ample volume did not exceed 10% of the volume of TRIZOL reagent. After homogenization, total RNA was isolated, treated with DNase I, and quantified by spectrophotometry according to the manufacturers’ protocols. Purified total RNA (1 µg) was used as a template for complementary DNA (cDNA) synthesis using Moloney murine leukemia virus (M‐MLV) reverse transcriptase (Thermo Fisher Scientific, CA) according to the manufacturer's instructions.

Total RNA from cumulus cells was isolated using TRIZOL reagent according to the manufacturer’s directions. Purified RNA was treated with DNase I and quantified by spectrophotometry. Purified total RNA (1 µg) was used as a template for cDNA synthesis using M‐MLV (Promega, CA) according to the manufacturer’s instructions.

All reverse transcriptase reactions included no‐template controls. RT‐qPCR was performed using an SYBR Green master mix (DRR420A, TaKaRa, Dalian, China) and an ABI PRISM 7500 Sequence Detection System (Applied Biosystems, CA). All reactions were performed in triplicate. RT‐qPCR conditions were as follows: 95°C for 2 min; and 40 cycles of 95°C for 15 s and 60°C for 1 min. The $2^{-▵▵C_{t}}$ method was used to determine the gene expression level (Livak & Schmittgen, 2001). Porcine β‐actin was selected as an internal control for mRNA. *T* tests were used to evaluate expression differences. All primers were designed using Primer 5.0 and are described in Table 1.

**Table 1:** Primers for genes

Gene	Primer sequence (5′‐3′)	Size (bp)	Annealing temperature (°C)
*CYP11A1*‐forward	GAGCAGGGAGAGTAGCAGTG	196	60
*CYP11A1*‐reverse	ACCAGGAGAGGGGATCTCAC		
*HSD17B4*‐forward	CTTCTACGGGCGTGTGG	297	60
*HSD17B4*‐reverse	TCCCTCAGAATTCCAGCATTG		
*3B‐HSD*‐forward	CAGCATAGAGGTGGCTGGAC	278	60
*3B‐HSD‐*reverse	TGGAGTTGTGTGTCAGGACG		
*StAR*‐forward	GGACGAGGTGCTGAGTAAAGT	161	60
*StAR*‐reverse	TCTGCAGGATCTTGATCTTCTTG		
*NRDR*‐forward	GCCGTCAACCCATTCTTTGG	109	60
*NRDR*‐reverse	GCACCACTGCCTTTGTCATC		
*β‐actin*‐forward	CAAGGCCAACCGTGAGAAGA	309	60
*β‐actin*‐reverse	TTCTCCTTGATGTCCCGCAC		

### 2.7 Western blot

Ovaries were lysed with radioimmunoprecipitation buffer (50 mM Tris‐HCl, pH 7.4; 150 mM NaCl; 1% TritonX‐100; 1% sodium deoxycholate; and 0.1% sodium dodecyl sulfate [SDS]) containing 1 mM phenylmethanesulfonyl fluoride (PMSF). The protein concentration of each group was determined using the BCA assay reagent (Vigorous Biotechnology, Beijing, China) according to the manufacturer's recommendations. An equal amount of protein (50 μg) was electrophoresed on 11% SDS polyacrylamide gel and transferred to a polyvinylidene difluoride (PVDF) membrane (Bio‐Rad Laboratories, Hercules, CA). The membrane was blocked with 5% (w/v) nonfat dry milk in 0.05 M Tris‐buffered saline (TBS; pH 7.4) for 3 hr. The membrane was then incubated with *NRDR* antibody (1:2,000, Abcam, Cambridge, UK) and internal control β‐actin antibody (1:2,000, Ambion, Austin, TX) overnight at 4°C. The PVDF membrane was then washed three times for 30 min in TBST (0.1% Tween‐20 in TBS) and incubated for 2 hr with horseradish peroxidase‐conjugated goat antirabbit IgG or horseradish‐peroxidase‐conjugated goat antimouse IgG (1:5,000, Zhongshan, Beijing, China). After washing for 30 min with three changes of TBST, Pierce™ ECL 2 Western Blot Substrate (Thermo Fisher Scientific, CA) was added to the membrane. The relative intensity of each blot was assessed and analyzed using AlphaImager 2200. The intensity values pertaining to each group were normalized against the OD of β‐actin corresponding to the same group within a single membrane and expressed in terms of the mean ± *SEM* of three independent experiments.

### 2.8 Immunofluorescence assay

Adult pig ovaries were embedded in paraffin and cut into 5‐μm sections. IFA of ovary sections was performed using methods described previously (Li et al., 2014). Antibodies against *NRDR* (1:100, Abcam, CA) were added to the sections and incubated at 4°C for 12 hr. After washing, sections were incubated with the goat antirabbit IgG (H + L) highly cross‐adsorbed secondary antibody, Alexa Fluor Plus 555 (1:500; Invitrogen, CA) at room temperature for 3 hr. For the NC, sections were incubated with a rabbit IgG antibody following the same abovementioned procedures. Sections were then incubated with 4′,6‐diamidino‐2‐phenylindole (DAPI) for 10 min. Slides were viewed under a microscope (Leica Microsystems, Cambridge, UK) and photographed.

### 2.9 Radioimmunoassays

Transfected cumulus cells (1 × 10^5^ cells/well in 24‐well plates) were cultured in DMEM with 10% fetal bovine serum for 24 hr. Cell medium was then replaced with serum‐free DMEM (1 ml/well), and cells were incubated for an additional 12 hr before harvesting. The medium and cells were both collected for progesterone (P4) and estradiol (E2) determinations. Experiments were performed six times. P4 and E2 were analyzed using radioimmunoassay (RIA) reagents provided by the Beijing North Institute Biological Technology (Beijing, China), which have been validated for use in cell culture media (Liu et al., 2015; Yu et al., 2016), according to the manufacturer’s recommendations. The minimum detectable concentrations were 2 pg/ml for E2 and 0.2 ng/ml for P4. For each RIA, the intra‐ and interassay coefficients of variation were less than 15% and 10%, respectively.

### 2.10 Detecting and genotyping polymorphism sites

Genomic DNA was extracted from ear tissue samples using a tissue and cell genomic DNA purification kit (Tiangen, Beijing, China) according to the manufacturer’s instructions. A pooled DNA sample was prepared by mixing equal amounts of DNA (50 ng per sample) from 20 randomly selected pigs. Primers were designed using Primer 5.0 to detect the SNPs of *DHRS4* as shown in Table 2. PCR assays were performed in a total volume of 20 µl containing 50 ng of template DNA according to previously described methods (Hou et al., 2010). Matrix‐assisted laser desorption/ionization time of flight mass spectrometry (MALDI‐TOF MS; Sequenom MassARRAY) was used to genotype 234 individuals by Beijing Compass Biotechnology Co., Ltd. (Beijing, China).

**Table 2:** Primers for *DHRS4* gene

Gene	Primer sequence (5′‐3′)
*DHRS4*1‐forward	TCCCTCCTTGGCTATCTGCT
*DHRS4*1‐reverse	AGCCAGGACTAGTCTCCCTG
*DHRS4*2‐forward	GAGTGTGTGCTGTCTTCGGA
*DHRS4*2‐reverse	GCCTTGAATCTTCCCTGCCT
*DHRS4*3‐forward	AGAGAGTCAGGGAGCCAGAG
*DHRS4*3‐reverse	GACCCTAAGGGGCATTTGCT
*DHRS4*4‐forward	GGTCAAGAGTTGCTGGCTCT
*DHRS4*4‐reverse	AGCAGATAGCCAAGGAGGGA
*DHRS4*5‐forward	CTGATGGGGAAATGCCTGGT
*DHRS4*5‐reverse	CCTGTGACCATGGGAACCTC
*DHRS4*6‐forward	GCCAGGCAGTTCACCCTAAT
*DHRS4*6‐reverse	AAGTGCTGGAACAACCCCAA
*DHRS4*7‐forward	CCTAACCTGGGGAGGAGAAGA
*DHRS4*7‐reverse	TGCAGTCAGAGTGGAAACCC

### 2.11 Statistical analysis

All expression and hormone experiments were independently performed three or more times. All data were analyzed using one‐way analysis of variance followed by Student’s *t* test. The values are presented as the mean ± *SEM*. Statistical analysis was performed using SPSS 10.0 (SPSS Inc., IL). A value of *p* < 0.05 was considered to be statistically significant.

The genotypic and allelic frequencies of each SNP were calculated using PopGene 3.2 software. Association analysis between SNPs and reproduction traits was performed using the general linear model (GLM) procedure of SAS 9.2 statistical software with the following fixed effect model:

$\;\;\;y=\mu +g_{i}+m_{k}+e$where *y* represents the phenotype records of reproduction traits; μ is the overall mean; gi is the fixed effect of the genotype; mk is the fixed effect of month‐old; and *e* is the random error (Zhou et al., 2017). Statistics are presented as probability values and least squares means ± standard error. The thresholds for statistical significance were *p < 0.05, **p < 0.01, and ***p < 0.001.

## 3 RESULTS

### 3.1 *NRDR* expression in pig cumulus granulosa cells

**Figure 1:**
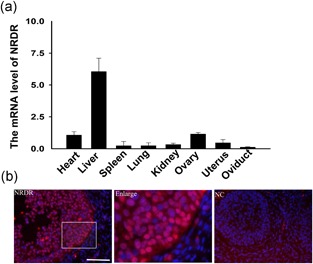
*NRDR* expression in pig ovary. (a) *NRDR* mRNA levels in different pig tissues. The experiments were repeated at least three times and normalized to the respective control. Data are shown as the means ± *SEM*. (b) Expression of *NRDR* in pig ovary using immunofluorescence assay. Red staining represents *NRDR*, and DAPI nuclear counterstaining (DNA) is blue. Bar represents 50 μM. mRNA: messenger RNA; *NRDR*: NADPH‐dependent retinol dehydrogenase/reductase; NC: negative control; *SEM*: standard error of mean [Color figure can be viewed at wileyonlinelibrary.com]

Messenger RNA (mRNA) expression levels for *NRDR* were initially detected in different pig tissues using real‐time quantitative polymerase chain reaction (RT‐qPCR). The *NRDR* mRNA level was highest in the liver. In the reproductive system, *NRDR* expression was high in the ovaries and uterus as shown in Figure 1a. Furthermore, *NRDR* expression was confirmed in the ovary by immunofluorescence assay (IFA). As shown in Figure 1b, *NRDR* was present in all granulosa cells, and no *NRDR* signal was observed in the corpus luteum or stromal cells. These results suggested that *NRDR* is involved in the regulation of steroid hormone synthesis and cell proliferation in cumulus granulosa cells.

### 3.2 High *NRDR* expression is present in pig breeds that have low levels of E2

**Figure 2:**
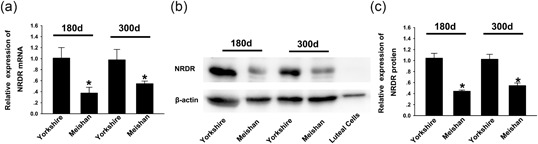
Yorkshire pig ovaries have higher *NRDR* expression than Meishan pig ovaries. (a) RT‐qPCR analysis of *NRDR* mRNA level in Yorkshire and Meishan pig ovaries on postnatal days 180 and 300. The experiments were repeated at least three times and normalized to their respective control. Data are shown as the mean ± *SEM* (n ≥ 3). *p < 0.05. (b) NRDR protein levels in Yorkshire and Meishan pig ovaries were measured by WB. Luteal cells from Yorkshire pigs served as a negative control. (c) Quantification of NRDR protein levels in Yorkshire and Meishan pig ovaries. The experiments were repeated at least three times and normalized to their respective control. Data are presented as the means ± *SEM* (n ≥ 3). *p < 0.05, (ANOVA). (180 days, postnatal day 180; 300 days, postnatal Day 300). ANOVA: Analysis of variance; mRNA: messenger RNA; NRDR: NADPH‐dependent retinol dehydrogenase /reductase; RT‐qPCR: real‐time quantitative polymerase chain reaction; *SEM*: standard error of mean; WB: Western blot

Previous studies have shown that a significant difference exists in the reproductive ability between Meishan and Yorkshire pigs (Hunter et al., 1996; Hunter, Faillace, & Picton, 1994; Miller, Picton, Craigon, & Hunter, 1998; Sun et al., 2011). In addition, the level of E2 in the follicular fluid of Meishan pigs is significantly higher than that in Yorkshire pigs (Hunter, Biggs, & Faillace, 1993; Miller et al., 1998). Therefore, the difference of *NRDR* expression in Yorkshire and Meishan pig ovaries in estrus at two time points after sexual maturity (postnatal Days 180 and 300) was detected by RT‐qPCR and western blot to elucidate the relationship between NRDR and reproductive ability. Higher levels of both *NRDR* mRNA and protein were expressed in Yorkshire pig ovaries compared with Meishan pig ovaries (Figure 2a‐c), that is, *NRDR* was overexpressed in pig breeds with lower levels of E2 and reproductive ability. The above results suggested that *NRDR* may inhibit E2 synthesis in ovaries and that *NRDR* has a relationship with the reproductive ability of pigs.

### 3.3 *NRDR* inhibits E2 synthesis in pig cumulus granulosa cells

**Figure 3:**
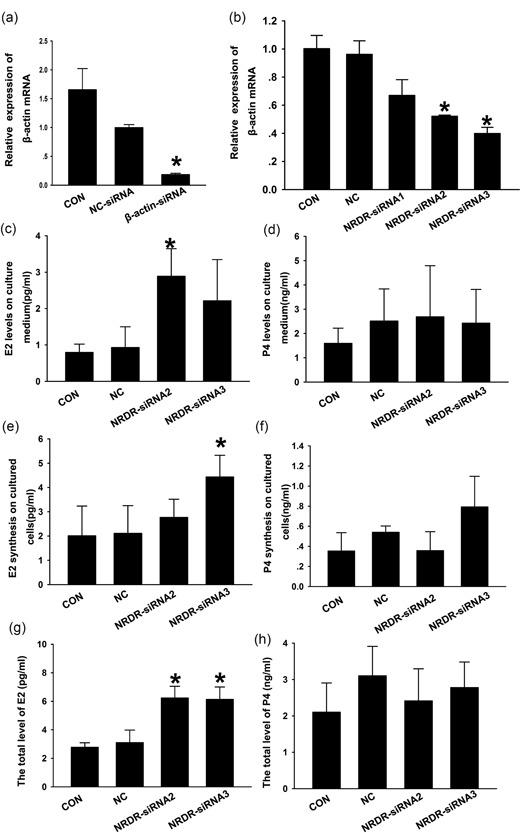
Effect of *NRDR* on E2 synthesis in pig cumulus granulosa cells. (a) Quantification of intracellular β‐actin mRNA levels in pig cumulus granulosa cells 24 hr after transfection with β‐actin siRNA. (b) Quantification of intracellular *NRDR* mRNA levels in pig cumulus granulosa cells 24 hr after transfection with *NRDR* siRNAs. (*n* = 3) *p < 0.05 versus NC‐siRNA (*t* test). (c‒f) Estradiol (c and e) and progesterone d and f were measured by radioimmunoassay in media c and d and in pig cumulus granulosa cells e and f after transfection with *NRDR* siRNA. (g‒h) Total levels of estradiol (g) and progesterone h in the media and cells were summed. Cells and medium were collected 36 hr after transfection. Data are shown as the means ± *SEM* (*n* = 6). *p < 0.05 versus NC‐siRNA or control (t test). CON: control, no treatment; mRNA: messenger RNA; NC: negative control; NRDR: NADPH‐dependent retinol dehydrogenase/reductase; *SEM*: standard error of mean; siRNA: small‐interfering RNAs

The effects of *NRDR* on steroid hormone production were determined in cultured pig cumulus granulosa cells using *NRDR* small‐interfering RNAs (siRNAs). The expression of *NRDR* was inhibited with siRNA, and β‐actin siRNA was used as a positive control (Figure 3a). *NRDR*‐siRNA2 and *NRDR*‐siRNA3 decreased *NRDR* mRNA levels by approximately 50–60% in cultured cells 24 hr after transfection similar to the levels of the positive control (Figure 3b). Inhibition of *NRDR* expression caused a twofold increase of E2 levels in the medium compared to the negative control (NC)‐siRNA (Figure 3c). However, P4 levels in the medium did not change significantly after *NRDR* inhibition (Figure 3d). To assay the effect of *NRDR* on E2 synthesis in cultured cells, E2 levels in the cultured cells were measured after *NRDR* knockdown. NRDR siRNAs had a significant effect on E2 synthesis in cultured cells (Figure 3e), but no significant changes in P4 levels were observed in cells after *NRDR* inhibition (Figure 3f). Summation of the total levels of E2 and P4 in the media and cells showed that both siRNAs significantly affected E2 level (Figure 3g) but did not affect P4 level (Figure 3h).

### 3.4 *NRDR* affects enzymes involved in E2 synthesis

**Figure 4:**
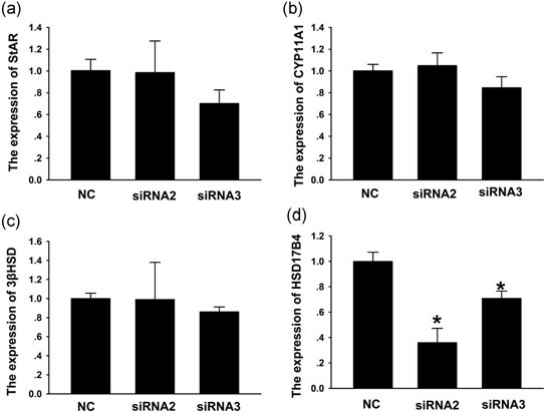
Effect of *NRDR* inhibition on enzymes involved in E2 synthesis. (a–d) Gene expression levels of the key enzymes involved in E2 synthesis were analyzed in pig cumulus granulosa cells 24 hr after cells were transfected with *NRDR* siRNAs. The enzymes included (a) steroidogenic acute regulatory protein (StAR), (b) cytochrome P450 family 11 subfamily A member 1 (cyp11A1), (c) 3‐β‐hydroxysteroid dehydrogenase (3β‐HSD), and (d) 17β‐hydroxysteroid dehydrogenase 4 (HSD17B4). Data are shown as the mean ± *SEM* (*n* = 3). *p < 0.05 versus NC (*t* test). NC: negative control; NRDR: NADPH‐dependent retinol dehydrogenase/reductase; *SEM*: standard error of mean; siRNA: small‐interfering RNA

The influence of *NRDR* inhibition on the mRNA levels of the following enzymes involved in E2 synthesis was determined: steroidogenic acute regulatory protein (*StAR*), cytochrome P450 family 11 subfamily A member 1 (*cyp11A1*), 3B‐hydroxysteroid dehydrogenase (3B‐HSD), and 17B‐hydroxysteroid dehydrogenase 4 (*HSD17B4*). Inhibition of *NRDR* with siRNAs significantly downregulated HSD17B4 mRNA levels (Figure 4d) but did not significantly affect *StAR*, *cyp11A1*, and *3β‐HSD* mRNA levels (Figure 4a–c). These results showed that *NRDR* affects E2 synthesis in pig cumulus granulosa cells.

### 3.5 *NRDR* does not affect viability and proliferation of pig cumulus granulosa cells

**Figure 5:**
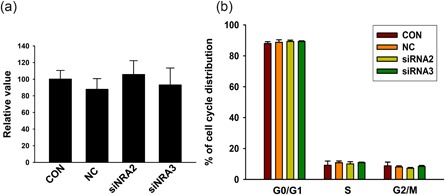
Effects of *NRDR* on cell viability and cycle in pig cumulus granulosa cells. (a) Cell viability was measured by fluorescence chemistry using a CCK8 kit after transfection with NRDR siRNAs. (b) Cell cycle was analyzed by flow cytometry in pig cumulus granulosa cells 48 hr after transfection with *NRDR* siRNAs. Cell cycle ratios were analyzed using approximately 20,000 cells per sample. Data are shown as the means ± *SEM* (*n* = 3 per group). CCK: cell counting kit; NC: negative control; NRDR: NADPH‐dependent retinol dehydrogenase/reductase; *SEM:* standard error of mean; siRNA: small‐interfering RNA [Color figure can be viewed at wileyonlinelibrary.com]

To determine if changes in estradiol levels were due to changes in cell viability or proliferation, the influence of NRDR siRNAs on viability and proliferation of pig cumulus granulosa cells was determined using a cell counting kit‐8 (CCK8) kit and flow cytometry. Inhibition of *NRDR* with siRNA had no effect on viability and proliferation of pig cumulus granulosa cells as shown in Figure 5a,b.

### 3.6 Effects of hCG and PMSG on *NRDR* expression in pig cumulus granulosa cells

**Figure 6:**
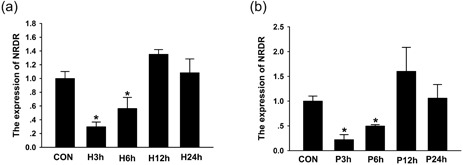
PMSG and hCG inhibit *NRDR* expression in pig cumulus granulosa cells. Changes in *NRDR* mRNA levels over time after treatment with (a) 10 IU/ml hCG or (b) 10 IU/ml PMSG. Results are shown as the means ± *SEM* of three independent experiments conducted using triplicates and normalized to control treatment, *p < 0.05 (*t* test). Con: control; hCG: human chorionic gonadotropin; mRNA: messenger RNA; NRDR: NADPH‐dependent retinol dehydrogenase/reductase; PMSG: pregnant mare serum gonadotropin; *SEM*: standard error of mean

As human chorionic gonadotropin (hCG) and pregnant mare serum gonadotropin (PMSG) are key hormones regulating E2 synthesis in pig cumulus granulosa cells, cultured cells were treated with 10 IU/ml hCG or PMSG for 0 (CON), 3, 6, 12, and 24 hr (Faerge et al., 2006; Hu et al., 2011; G. S. Lee, Kim, Hwang, & Hyun, 2008). *NRDR* mRNA levels were assayed to identify the upstream factors affecting *NRDR*. At 3 and 6 hr after hCG and PMSG treatment, *NRDR* mRNA levels were significantly downregulated by approximately 75% (Figure 6a,b). Human CG and PMSG treatment had no effect on *NRDR* expression after 12 and 24 hr of treatment.

### 3.7 Relationship between reproductive traits and *the NRDR gene (DHRS4)* polymorphisms

The relationship between reproductive traits and *the NRDR gene (DHRS4)* polymorphisms was analyzed. Analysis of the intron and exon sequences of pooled DNA samples from randomly selected pigs chosen from a crossbred population derived from RiZhaoDaBai pigs revealed seven SNPs in the *DHRS4* gene. Of the seven SNPs, three SNPs were successfully genotyped using Sequenom MassARRAY (Table 3). Among these three SNPs, two were significantly associated with changes in reproductive traits (Table 4).

**Table 3:** Genotype and allele frequencies of the three SNPs identified in *DHSR4*

SNPs	Allele frequency		Genotype frequency		
rs342446613	A	T	AA	TT	TA
	0.169	0.831	0.028 (6)	0.688 (161)	0.286 (67)
rs701332503	C	T	CC	TT	TC
	0.432	0.568	0.158 (37)	0.295 (69)	0.547 (128)
rs326982309	A	T	AA	TT	TA
	0.844	0.156	0.701 (164)	0.013 (3)	0.286 (64)

*Note*. SNP: single‐nucleotide polymorphism.

**Table 4:** Association of rs341891833 and rs326982309 SNPs with reproductive traits in RZDB pigs

		Primiparity			Multiparity		
SNPs	Genotype (sample size)	TNB	TLW	ABW	TNB	TLW	ABW
rs701332503	CC(37)	8.67 ± 0.43	11.67 ± 0.69	1.38 ± 0.04	10.95 ± 0.38	14.07 ± 0.64	1.35 ± 0.05
	TT(69)	9.43 ± 0.32	13.41 ± 0.51	1.42 ± 0.03	9.56 ± 0.25**	13.91 ± 0.45	1.43 ± 0.04
	CT(128)	9.08 ± 0.23	12.73 ± 0.37	1.40 ± 0.02	9.35 ± 0.20***	13.79 ± 0.33	1.42 ± 0.03
rs326982309	AA(164)	9.57 ± 0.29	12.83 ± 0.32	1.39 ± 0.02	9.96 ± 0.17	14.20 ± 0.27	1.44 ± 0.02
	TT(3)	9.33 ± 2.03	12.00 ± 2.15	1.31 ± 0.15	10.50 ± 1.16	15.58 ± 1.75	1.48 ± 0.14
	TA(64)	8.95 ± 0.47	13.15 ± 0.51	1.49 ± 0.03*	10.22 ± 0.26	13.93 ± 0.40	1.37 ± 0.03

*Note*. Data are reported as the mean ± *SE*. ABW: average birth weight; RZDB: RiZhaoDaBai; TNB: total number of piglets born; TLW: total litter weight of sows ; *SE*: standard error.

*p < 0.05, **p < 0.01, ***p < 0.001.

SNP rs701332503 (chr7:75245594 C > T) was a variant located in the coding region of *DHRS4*. This SNP was significantly associated with changes in the total number of piglets born (*p* < 0.01; Table 4) during multiparity. Animals with mutation genotype CC in rs341891833 had more piglets than those with genotypes TT and CT.

SNP rs326982309 (chr7: 75253401 A > T) was an intron variant located in the intron region of NM_214019.2 of *DHRS4*. This SNP was significantly associated with changes in ABW (*p* < 0.05; Table 4) during primiparity. Animals with mutation genotype TA in rs326982309 had higher birth weights than those with genotypes TT and AA.

## 4 DISCUSSION

The present work showed that *NRDR* is expressed in pig cumulus granulosa cells and is involved in the regulation of E2 synthesis. Previous studies have reported that *NRDR* is related to human cervical cancer, breast cancer, and other cancer tissues (Korkola et al., 2007; X. H. Song et al., 2007), but studies showing the systematic expression of *NRDR* in different tissues of the pig have not been published. *NRDR* was expressed at higher levels in the ovary compared to other tissues, except the liver. *NRDR* was specifically expressed in cumulus granulosa cells (shown by immunofluorescence assay), which corresponds to the area of steroid hormone synthesis in pig ovary (Yamashita et al., 2003). Studies have shown that the level of E2 in Meishan pig follicular fluid is significantly higher than that in Yorkshire pigs (Miller et al., 1998). Different expression levels of *NRDR* exist between Yorkshire and Meishan pig ovaries. The higher expression of *NRDR* in pig breeds with low levels of E2 is related to the *NRDR* function of inhibiting E2 synthesis.

*NRDR* is overexpressed in swine breeds with high androstenone levels (Grindflek, Berget, Moe, Oeth, & Lien, 2010; Leung, Bowley, & Squires, 2010; Moe et al., 2007). Several members of the SDR family are important in catalyzing an essential step in the biosynthesis of all classes of active steroid hormones (Penning, 1997). The present results showing that *NRDR* inhibits E2 synthesis in pig cumulus granulosa cells are consistent with the above reports. *NRDR* catalyzes the reduction of 3‐keto‐C19/C21‐steroids into corresponding 3B‐hydroxysteroids (Matsunaga et al., 2008). In a rabbit, pig, dog, and human, DHRS4 exhibits high reductase activity towards aromatic ketones and α‐dicarbonyl compounds as well as low dehydrogenase activity towards some alcohols (Endo et al., 2007; Usami et al., 2003). Studies in hamsters, pig, mice, and rats have demonstrated that estradiol, estrone (interconvertible metabolite of estradiol), and their catechol metabolites exist in the kidney, uterus, ovary, and mammary glands (Prater, Horton, & Thompson, 2015; Yager, 2015). According to these results, *NRDR* may play an important role as a reductase in the interconversion between estrone and estradiol.

StAR, cyp11A1, 3β‐HSD, and HSD17Β4 are key enzymes in steroid hormone biosynthesis (Fukami, Homma, Hasegawa, & Ogata, 2013; Robic, Faraut, & Prunier, 2014). The present study evaluated the expression of these enzymes after *NRDR* was inhibited and found that only *HSD17Β4* expression was downregulated. Moreover, *HSD17Β4* catalyzes the last steps in the formation of androgens and estrogens (Payne & Hales, 2004). *NRDR* may affect E2 biosynthesis at the last steps by inhibiting *HSD17B4* expression without affecting upstream enzymes. *HSD17Β4* is expressed as the predominant dehydrogenase in several pig tissues (Kaufmann, Carstensen, Husen, & Adamski, 1995). *HSD17Β4* has also been shown to efficiently inactivate estrogens in several tissues due to the preference for steroid oxidation (Adamski et al., 1995; Breitling, Marijanović, Perović, & Adamski, 2001). Similarly, when *NRDR* was inhibited in the present study, *HSD17Β4* expression was downregulated, and E2 biosynthesis was upregulated. The change in *HSD17B4* expression after *NRDR* inhibition indirectly suggested that *NRDR* affects E2 biosynthesis. In addition, both *NRDR* and HSD17Β4 are expressed at higher levels in high androgen pigs, supporting the idea that *NRDR* participates in steroid hormone biosynthesis (Grindflek et al., 2010; Leung et al., 2010; Moe et al., 2007).

The present study revealed that inhibition of *NRDR* upregulates the level of E2. To determine whether the increase in E2 was caused by increased proliferation of pig cumulus granulosa cells or upregulation of E2 synthesis, the effects of *NRDR* on the cell cycle were determined. No effect of *NRDR* on proliferation of pig cumulus granulosa cells was observed, suggesting that *NRDR* affects E2 synthesis. This result was in agreement with a previous study showing that the level of steroid hormones has no direct relationship on the proliferation of cumulus granulosa cells (Elis et al., 2015). The effects of hCG and PMSG on *NRDR* expression were also examined. Both hCG and PMSG significantly inhibited *NRDR* expression at 3 hr, which correlated with the effects on other genes immediately (i.e., 1 and 3 hr) after LH/hCG treatment (Carletti & Christenson, 2009; Cheng, Fang, Chang, Sun, & Leung, 2016; Park et al., 2004). These data suggested that hCG and PMSG are upstream regulators of *NRDR* involved in E2 synthesis.

Previous studies have shown that differential expression of alleles is quite common in mammals and that variations may contribute to phenotypic variability (Lo et al., 2003; Yan, Yuan, Velculescu, Vogelstein, & Kinzler, 2002). SNPs may affect the expression of *NRDR*. Because inhibition of *NRDR* increased E2 levels, we speculated that the level of E2 was higher in pigs with *NRDR* mutations. According to previous reports, high levels of E2 are unfavorable for embryo implantation and may lead to reduced number of piglets (Burghardt, Bowen, Newton, & Bazer, 1997; Geisert, Renegar, Thatcher, Roberts, & Bazer, 1982). The present results showed that pigs with SNPs in the *DHRS4* gene had fewer piglets born, which was in agreement with previous reports. Furthermore, the present SNP results may be useful for marker‐assisted and genomic selection strategies for genetic improvement programs in pigs. However, further studies using methods, such as Cas9 editing and gene silencing analysis, are needed to determine the biological functions of these significant SNPs.

In summary, the present study demonstrated that *NRDR* is highly expressed in pig ovaries, specifically in cumulus granulosa cells. Functional studies showed that *NRDR* is involved in regulating E2 synthesis in pig cumulus granulosa cells but has no effect on the proliferation of pig cumulus granulosa cells. PMSG and hCG both downregulated the expression of *NRDR* in pig cumulus granulosa cells. The relationship between reproductive traits and polymorphisms in the *NRDR* gene (*DHRS4*) was also analyzed. The rs701332503 SNP was significantly associated with changes in the total number of piglets born during multiparity, and the rs326982309 SNP was significantly associated with changes in ABW during primiparity. Thus, these findings demonstrated that *NRDR* has an important role in pig steroid hormone biosynthesis. SNP analysis and further verification of the role of these polymorphisms may lead to the use of *NRDR* as a selective marker to improve pig reproduction.

## CONFLICTS OF INTEREST

The authors declare that they have no conflicts of interest.

## AUTHOR CONTRIBUTIONS

K. L., R. Z., Z. G., H. A., and Y. L. conceived the project and designed experiments; Y. L. and Y. Z. analyzed data; Y. Z. and Y. Y. collected samples; Y. L., W. L., and Y. Z. performed experiments; and Y. L. and Y. Y. wrote the manuscript. All authors read and approved the final manuscript.
